# Glycolysis and Fatty Acid Oxidation Inhibition Improves Survival in Glioblastoma

**DOI:** 10.3389/fonc.2021.633210

**Published:** 2021-03-29

**Authors:** Kelly J. McKelvey, Erica B. Wilson, Susan Short, Alan A. Melcher, Michael Biggs, Connie I. Diakos, Viive M. Howell

**Affiliations:** ^1^Bill Walsh Translational Cancer Research Laboratory, Faculty of Medicine and Health, The University of Sydney, St Leonards, NSW, Australia; ^2^Translational Neuro-Oncology, Leeds Institute of Medical Research at St James's, University of Leeds, Leeds, United Kingdom; ^3^Translational Immunotherapy, Division of Radiotherapy and Imaging, Institute for Cancer Research, London, United Kingdom; ^4^Department of Neurosurgery, North Shore Private Hospital, St Leonards, NSW, Australia; ^5^Department of Medical Oncology, Northern Sydney Cancer Centre, Royal North Shore Hospital, St Leonards, NSW, Australia; ^6^Northern Clinical School, Faculty of Medicine and Health, The University of Sydney, St Leonards, NSW, Australia

**Keywords:** glioblastoma, cancer metabolism, ranolazine, dichloroacetate, radiation, temozolomide

## Abstract

Glioblastoma (GBM) is the most aggressive adult glioma with a median survival of 14 months. While standard treatments (safe maximal resection, radiation, and temozolomide chemotherapy) have increased the median survival in favorable O(6)-methylguanine-DNA methyltransferase (MGMT)-methylated GBM (~21 months), a large proportion of patients experience a highly debilitating and rapidly fatal disease. This study examined GBM cellular energetic pathways and blockade using repurposed drugs: the glycolytic inhibitor, namely dicholoroacetate (DCA), and the partial fatty acid oxidation (FAO) inhibitor, namely ranolazine (Rano). Gene expression data show that GBM subtypes have similar glucose and FAO pathways, and GBM tumors have significant upregulation of enzymes in both pathways, compared to normal brain tissue (*p* < 0.01). DCA and the DCA/Rano combination showed reduced colony-forming activity of GBM and increased oxidative stress, DNA damage, autophagy, and apoptosis *in vitro*. In the orthotopic Gl261 and CT2A syngeneic murine models of GBM, DCA, Rano, and DCA/Rano increased median survival and induced focal tumor necrosis and hemorrhage. In conclusion, dual targeting of glycolytic and FAO metabolic pathways provides a viable treatment that warrants further investigation concurrently or as an adjuvant to standard chemoradiation for GBM.

## Introduction

Glioblastoma (GBM) is a biologically heterogeneous and uniformly fatal disease. Verhaak et al. (1) describe classical, mesenchymal, neural, and proneural subtypes of GBM based on an 840 gene signature. These have been adopted as clinically relevant molecular subtypes with different disease progression and prognosis. While brain cancer makes up 1.3% of all new cancer diagnoses in Australia, it accounts for 3.2% of cancer-related deaths (2). GBM accounts for 54% of brain cancer cases and has a 5-year survival rate <5% (3). Despite the recognized heterogeneity in GBM epigenetics, genetics, and transcription (4); histological patterns (5); immune infiltration (6); and metabolism (7), the current treatment for GBM remains maximal safe surgical resection, followed by external beam radiation therapy (RT) with concurrent and adjuvant temozolomide (TMZ) (8). This increases median overall survival from 14 months for TMZ-resistant O(6)-methylguanine-DNA methyltransferase (MGMT) promoter unmethylated GBMs to 21.2 months for TMZ-sensitive MGMT promoter methylated GBMs (9).

In addition to a uniform reliance of cancer cells on aerobic glycolysis, recent studies have demonstrated the dependence of GBM cells on fatty acid oxidation (FAO) (7). Human gliomas express the FAO enzymes medium-chain acyl-CoA dehydrogenase (MCAD), short-chain L-3-hydroxyacyl-CoA dehydrogenase (SCHAD), very-long-chain acyl-CoA dehydrogenase (VLCAD), hydroxyacyl-CoA dehydrogenase (HADH)/3-ketoacyl-CoA thiolase/enoyl-CoA hydratase (ECH), and carnitine palmitoyltransferase 1a (CPT1a) and demonstrate decreased oxygen consumption in the presence of an FAO inhibitor, etomoxir (10). Genomic, metabolomic, and functional analyses by Prabhu et al. (7) show that FAO is a key driver of progression from low-grade gliomas into GBM. In a syngeneic glioma model (oncogenic neural stem cells), etomoxir increased survival (10), whereas, the combination of etomoxir, glucose analog, and glycolytic inhibitor 2-deoxy-D-glucose (2-DG) led to metabolic lethality *in vitro* and increased median survival in MES93 mesenchymal GBM tumor-bearing mice (11). Ranolazine (Rano), a partial FAO inhibitor, has shown neuroprotective effects on healthy astrocytes and neurons in rodent cell cultures (12). Targeting FAO in GBM also shows promise in alleviating the immunosuppressive tumor microenvironment as FAO ameliorates the tolerogenic and immunosuppressive mechanisms of tumor myeloid-derived suppressor cells (MDSC) and reduces tumor growth in the syngeneic murine models of lung (3LL) and colon (MCA-38) cancer (13).

Glycolytic inhibitor dichloroacetate (DCA) induces apoptosis in GBM and putative GBM stem cells *in vitro* and *in vivo* (14) and has demonstrated clinical efficacy in a small cohort of patients with GBM (15). In addition, DCA sensitizes GBM to radiation (16) and has synergistic effects on GBM growth and survival induced by mitochondrial oxidative stress, when combined with mitochondrial inhibitor 4-[N-(S-penicillaminylacetyl)amino] phenylarsonous acid (17) and when inhibiting stem cell self-renewal in combination with metformin (18).

Glucose metabolism and reactive oxygen species (ROS) are bidirectionally linked, and cancer cells are metabolically adaptive to the changing oxygen and nutrient microenvironment (19). Therein, it was hypothesized that dual blockade of the glycolysis and FAO pathways would inhibit GBM tumor growth by increasing oxidative phosphorylation and enhancing chemoradiation-induced ROS. In the present study, we investigative the novel combination of the glycolytic inhibitor, DCA, and the partial FAO inhibitor, Rano, in two syngeneic immune-competent models of GBM, against “standard of care” chemoradiation.

## Materials and Methods

### Human Gene Expression Data

Gene lists for human glucose (#RAH49R3) and FAO metabolism (#RPKA3DE) were obtained from Taqman® Arrays (https://www.thermofisher.com). Gene expression and GBM subtype data from 48 primary GBM cell lines were acquired from the human glioblastoma cell culture (HGCC) Affymetrix Human Exon 1.0 ST Array (NCBI Gene Omnibus GSE72217; https://www.ncbi.nlm.nih.gov/geo/) (20). Classical, mesenchymal, proneural, and neural GBM subtyping was performed by HGCC (20) using the gene signatures described by Verhaak et al. (1). Data were analyzed using Gene Cluster 3.0 for Windows, Department of Genetics, Stanford University, CA, USA (21) and TreeView 3.0 beta1 for Windows, Department of Genetics, Stanford University, CA, USA (22) with the Pearson's correlation proximity-based hierarchical clustering. The Cancer Genome Atlas (TCGA; https://www.cancer.gov/tcga) GBM, TCGA normal, and the Genotype-Tissue Expression (GTEx; https://www.gtexportal.org/home/) data box plots were acquired through the Gene Expression Profiling Interactive Analysis (23) (GEPIA; available at http://gepia.cancer-pku.cn/). Data are expressed as the log_2_ fold change (FC) [defined as median (tumor) – median (normal)].

### Cells

Murine glioma Gl261 cells were donated by Géza Safrany (Frederic Joliot-Curie National Research Institute for Radiology and Radiohygiene, Budapest, Hungary) and CT2A by Tomas Seyfried (Boston College, Boston, MA, USA). U-87 MG, U-251 MG, and T98G from the CellBank Australia, Westmead, NSW, Australia. Cell lines were authenticated by the Satellite Tandem Repeat profiling (Garvan Institute of Medical Research, Sydney, NSW, Australia) and mycoplasma negative by the MycoProbe® Mycoplasma Detection Kit (R&D Systems, Inc., Minneapolis, MN, USA). Cells were cultured in Dulbecco's modified Eagle's medium (DMEM) containing 10% v/v fetal bovine serum (FBS) in a humidified incubator with 5% CO_2_ at 37°C with 60% relative humidity.

### Treatments

Drugs used in this study were DCA (sodium DCA #347795; Sigma-Aldrich, St. Louis, MO, USA), TMZ (#T2577; Sigma-Aldrich St. Louis, MO, USA), and Rano (Rano dihydrochloride #R6152; Sigma-Aldrich St. Louis, MO, USA) dissolved in complete media for *in vitro* studies and in sterile water for *in vivo* studies at stock concentrations of 40, 5, and 10 mg/ml, respectively. X-ray radiation was delivered using the Small Animal Radiation Research Platform (SARRP; Xstrahl Inc., Suwanee, GA, USA) at a dose rate of 2.97 Gy/min as described earlier (24). In all experiments, the drug treatment preceded irradiation by 1 h to enable the drugs to act on their respective cellular targets. The cells were detached with 0.5% v/v and trypsin/0.2% v/v ethylenediaminetetraacetic acid in phosphate-buffered saline (PBS) and counted by MUSE® cell count and viability assay (Luminex Corp., Austin, TX, USA) according to the instructions of the manufacturer.

### Cell Survival

Cells were plated at 8,000 cells per well in a 96-well plate with 100 μl of medium per well, which were left overnight to equilibrate, and assessed for confluency (%) after 72 h following the drug treatments. Five images per 96 wells (×10 objective) were acquired using the IncuCyte® Live Imaging System (IncuCyte® Software (v2019B), Sartorius, Gottingen, Germany) and analyzed using the imaging analysis software. Six wells were used per treatment per experimental replicate.

### Energy Metabolism Assays

Cells were plated at 40,000 cells per well in a 96-well plate with 200 μl of medium per well and left overnight to equilibrate. The Glycolysis (extracellular acidification; ab197244; Abcam, Cambridge, UK), FAO Complete (ab222944; Abcam, Cambridge, UK), and Extracellular Oxygen Consumption (ab197243; Abcam, Cambridge, UK) assays were performed as per the instructions of the manufacturer and detected using the dual-read time-resolved fluorescence (FLUOstar Omega; BMG LABTECH, Ortenberg, Germany). For FAO metabolism, cells were glucose deprived overnight. Carbonyl cyanide 4-(trifluoromethoxy) phenylhydrazone (FCCP; 2.5 μm), etomoxir (40 μm), and antimycin A (1 μm) were used as positive and negative controls where appropriate. Data are expressed as percentage effect of Lifetime (μs; **Equation (1)**) for the treatment relative to the untreated control.

(1) $$\begin{array}{l} Lifetime\;\left(\mu s\right)\;\left[T\right]=\left(D2-D1\right)/\text{ln }\left(\frac{W1}{W2}\right) \end{array}$$

where, W1 and W2 are the times for the dual measurement windows and D1 and D2 are the delay times prior to W1 and W2, respectively.

### Clonogenic/Colony Forming Unit Assay

Cells were seeded at 1,500 cells per well in 6-well plates with 2 ml of DMEM/10% v/v FBS. After 10 days, colonies were stained with crystal violet (0.5% w/v, 1:1 methanol:distilled water) and then imaged and quantitated using a vSpot® Spectrum ELISpot/FluoroSpot Reader System (Autoimmun Diagnostika Gmbh, Straßberg, Germany). Three wells were used per treatment per experimental replicate.

### Functional Assays

Cells were plated at 500,000 cells per T25 flask, equilibrated overnight, and then treated for 72 h. The Luminex® Oxidative Stress (ROS; Luminex Corp., Austin, TX, USA), DNA Damage [ataxia telangiectasia mutated (ATM)/histone 2A family member × (H2A.X)], Cell Cycle, Annexin-V, and Dead Cell, and Autophagy LC3 assays were carried out according to the instructions of the manufacturer. Cell events (1,000–5,000 assay dependent) were acquired and analyzed using the Guava® Muse® Cell Analyser (Luminex Corp., Austin, TX, USA).

### Western Blot

About 50 μg protein was loaded on Any kDa™ Mini-PROTEAN® TGX™ Precast protein gels and transferred to low-autofluorescence polyvinylidene fluoride (PVDF) membranes (Bio-Rad, Hercules, CA, USA). For autophagy Western blots, cells were treated with a 100 nM Bafilomycin A1 (B1793; Sigma-Aldrich St. Louis, MO, USA) 4 h before collection to accumulate LC3B in the cytoplasm. Primary antibodies were γH2A.X (1:1,000; ab11174; Abcam, Cambridge, UK), cyclin-dependent kinase 2 (Cdc2) (1:1,000; 28439S; Cell Signaling Technology, Danvers, MA, USA), cyclin B1 (1:1,000; 4138S; Cell Signaling Technology, Danvers, MA, USA), p21 (1:1,000; ab188224; Abcam, Cambridge, UK), Bax (1:1,000; ab3191; Abcam, Cambridge, UK), Bcl-2 (1:1,000; ab16904; Abcam, Cambridge, UK), caspase 3 (1:1,000; ab188224; Abcam, Cambridge, UK), SQSTM1/p62 (1:1,000; ab56416; Abcam, Cambridge, UK), LC3B (1:1,000; PM036; MBL International Corp., Woburn, MA, USA), and β-actin (1:10,000; A1978; Sigma-Aldrich St. Louis, MO, USA). Immunoblots were detected with 1:10,000 goat-anti-mouse DyLight™ 680 conjugated or donkey-anti-rabbit DyLight™ 800 conjugated using an Odyssey CLx Near-Infrared Fluorescence Imaging System (LI-COR Biosciences, Lincoln, NE, USA).

### *In vivo* Glioma Models

The murine survival study was reviewed, approved, and performed in accordance with the guidelines of the Northern Sydney Local Health District Animal Ethics Committee, Royal North Shore Hospital, St Leonards, NSW, Australia (Approval #RESP/17/205), which enforces the New South Wales Animal Research Act 1985.

Eight-week-old male C57Bl/6 mice (20–26 g) were provided by the Kearn's Animal Facility, Australia. Mice were housed in Allentown individually ventilated cages (3–5 per cage) with cellulose bedding under specific pathogen-free conditions. Enrichment was provided in the form of autoclaved ice block sticks or straws. Rooms were temperature-controlled (22°C) and were kept on a 12-h light/dark cycle (7:00/19:00 h) with a standard chow and water *ad libitum*.

Mice were inoculated with 1 × 10^5^/2 μl murine glioma Gl261 or CT2A cells using a stereotactic frame, a microinjection unit (David Kopf Instruments, Tujunga, CA, USA), and a 5 μl syringe with custom 32-G needle (Hamilton Company, Reno, NV, USA) into the right caudoputamen (striatum) at 2 mm in mediolateral, 0.1 mm in anteroposterior, 2.6 mm in dorsoventral bregma under isoflurane anesthesia (2% v/v per 1 L oxygen i.h.) as described earlier (6). Mice were randomly assigned into one of the eight treatment groups (six mice per group). Treatments were administered five times a week for 2 weeks commencing at day 7 postinoculation: TMZ (50 mg/kg/day i.p.), DCA (200 mg/kg/day i.p.), and Rano (50 mg/kg/day i.p.). External beam irradiation of 20 Gy/10 (i.e., 2 Gy per dose; RT) was performed using the Image-Guided SARRP (Xstrahl Inc., Suwanee, GA, USA) using a 5 × 5 mm collimator and 60° (30° to –30°) Arc beam at a dose rate of 3.71 Gy/min (24). For combination treatment groups, radiation was delivered 1-h post-drug administration.

Animal weight and well-being were assessed two times every week and euthanized by cardiac puncture under isoflurane anesthesia (2% v/v per 1 L oxygen i.h.) followed by cervical dislocation at the humane endpoint or a long-term survival (100 days postinoculation). No adverse events were encountered.

### Histopathological Analyses

Four-micron sections of paraffin-embedded tissues were stained with Mayer's H&E Y/erythrosine B staining, Ki67 (0.084 μg/ml; 12202S; Cell Signaling Technologies, Danvers, MA, USA), and γH2A.X (0.06 μg/ml; ab11174; Abcam, Cambridge, UK) as described earlier (6, 24) and terminal deoxynucleotidyl transferase dUTP nick end labeling (TUNEL) apoptosis by immunohistochemical staining [ab206386; Abcam, Cambridge, UK) with Mayer's hematoxylin nuclear stain. Slides were imaged using an Aperio XT slide scanner and captured using Aperio ImageScope (Leica Biosystems, Wetzlar, Germany), and five high-power images were assessed per sample (*N* = 4–6 brains per treatment group] using ImmunoRatio (Seinajoki, Finland) as described earlier (24).

### Statistical Analyses

Drug IC50 concentrations were calculated using a four-parameter response vs. drug concentration non-linear regression, and synergistic doses were calculated using the CompuSyn Software (Paramus, NJ, USA) (25, 26). To determine the statistical difference between treatments, the two-way ANOVA test along with the Tukey's multiple comparison test were performed for Annexin-V, DNA Damage, Oxidative Stress, and Cell Cycle MUSE® assays (Luminex Corp., Austin, TX, USA); and the one-way ANOVA test was performed with the Dunn's multiple comparison test for Autophagy LC3, Western blot fluorescent intensity, and CFU assays. Murine survival studies are expressed as Kaplan–Meier curves. All statistics were performed using the Prism version 8 for Windows (GraphPad Software Inc., San Diego, CA, USA), considering a significant *p*-value <0.05.

## Results

### Glucose and FAO Metabolism in GBM

**Figure 1:**
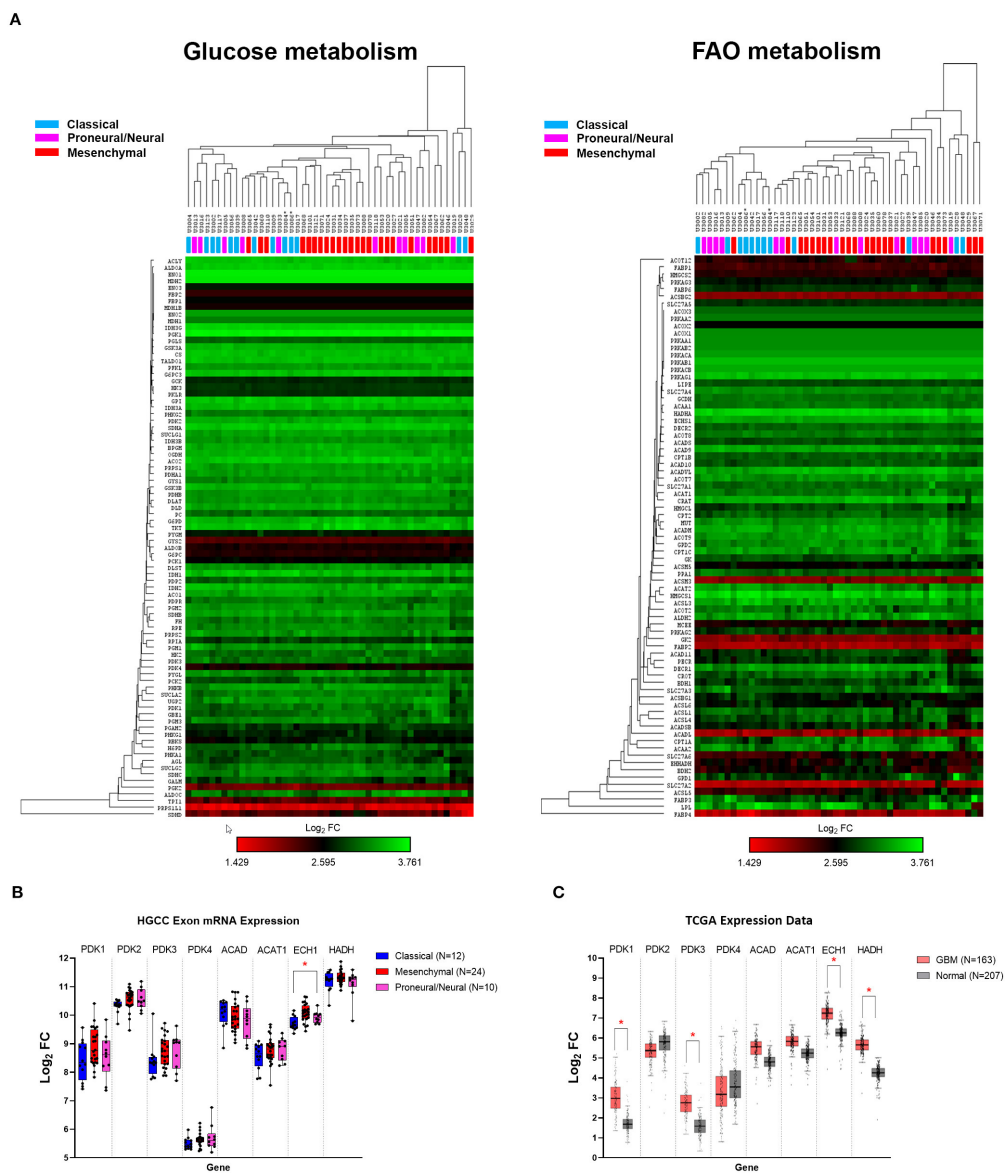
Human gene expression of glucose and fatty acid oxidation (FAO) pathways. **(A)** Heat map and hierarchical clustering of genes associated with the glucose and FAO metabolic pathways. **(B)** Human glioblastoma cell culture (HGCC) glioblastoma (GBM) subtype gene expression of selected targetable enzymes involved in glycolytic and β-oxidation pathways. **(C)** The Cancer Genome Atlas (TCGA) GBM vs. normal gene expression. Data are expressed as median ± interquartile range of log_2_ fold change [median (tumor) − median (normal)]. *p < 0.01 by the Kruskal–Wallis test with the Dunn's multiple comparison test.

Using the publicly available HGCC gene expression dataset, we investigated the glucose and FAO metabolic pathways in 48 GBM primary cell lines. Hierarchical clustering demonstrated that glucose and FAO metabolic pathways did not differ between the classical, mesenchymal, and proneural/neural GBM subtypes, offering a potentially wide-ranging therapeutic avenue (Figure 1A), compared to therapies targeting specific genetic mutations (e.g., EGFR and IDH1). This contrasts a recent report of 498 GBM IDH wildtype tumours which demonstrated increased glycolytic activity in the mesenchymal subtype (27).

Further examination showed that the expression of selected targetable enzymes in the glycolytic pathway, pyruvate dehydrogenase kinase (PDK) 1–4, and the FAO pathways, acetyl CoA dehydrogenase (ACAD), thiolase [acetyl-coenzyme A acetyltransferase (ACAT)], cronotase (ECH), and HADH, were similarly expressed across the GBM subtypes, except ECH was significantly higher in proneural/neural compared to classical tumors (*p* = 0.0022; Figure 1B). To confirm the role of PDKs and FAO enzymes in GBM, we compared the gene expression using the TCGA GBM and normal brain. The expression of PDK1, PDK3, ECH, and HADH were ~1.5-fold higher in the GBM tumors compared to the normal tissue (all *p* < 0.01; Figure 1C). These data confirm that targetable metabolic pathways exist within GBM.

From these results, we sought to investigate whether the combination of PDK inhibitor, DCA, and FAO enzyme inhibitor, Rano, can inhibit GBM cellular metabolism and cell growth and can increase survival in the syngeneic Gl261 and CT2A models of GBM.

### Gl261 and CT2A Demonstrate Different Sensitivities to Treatment

To determine the IC50 for drug and RT treatment, murine GBM cell lines were treated with a 2-fold increase in drug concentrations, and cell survival was assessed as either % confluency at 72 h for drug treatment or colony-forming units (CFUs) at 10 days for RT.

**Figure 2:**
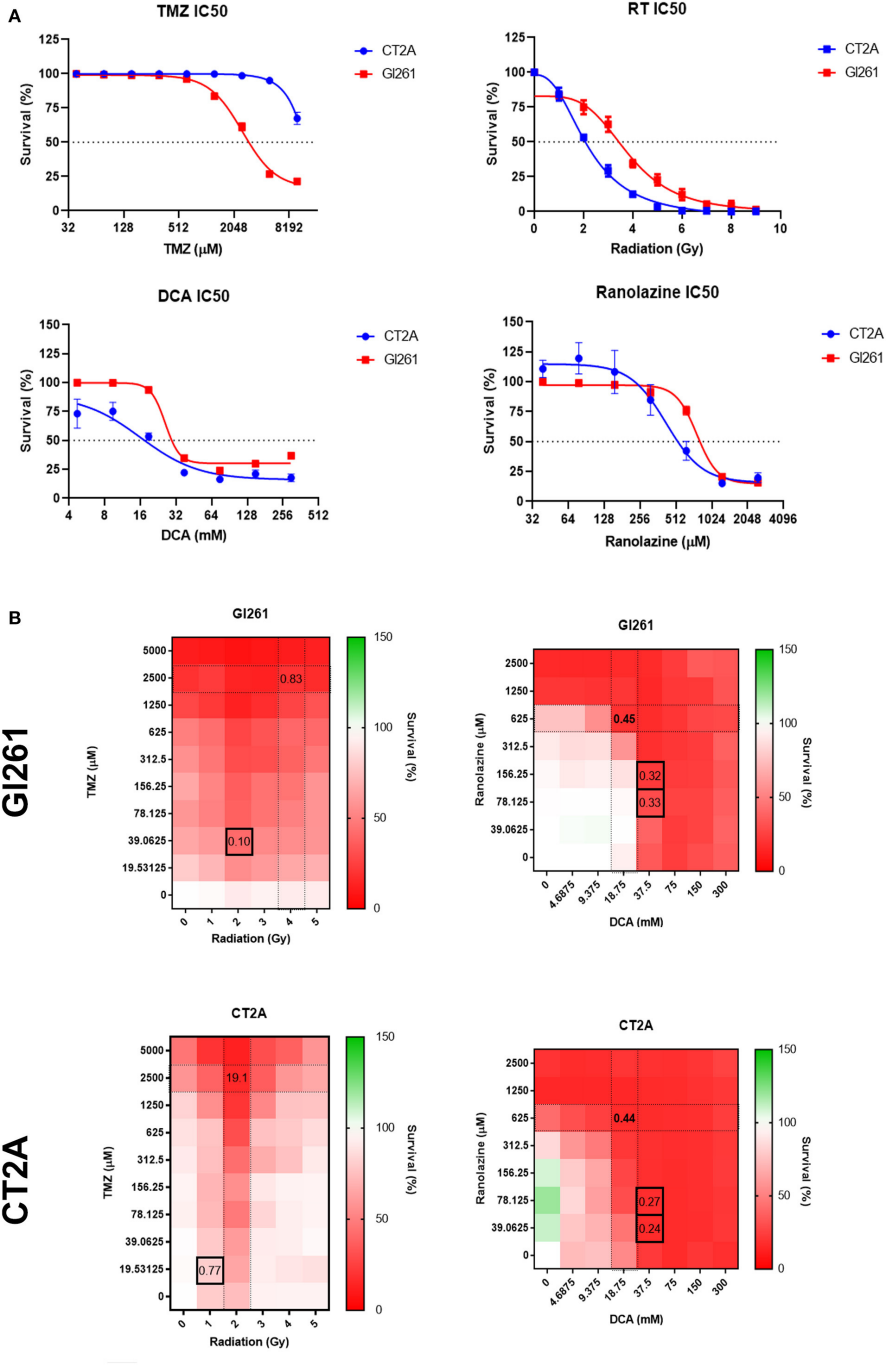
Sensitivity and survival of murine glioma cell lines to drug and radiation therapy (RT) treatment combinations. **(A)** Drug IC50s for temozolomide (TMZ), dicholoroacetate (DCA), and ranolazine (Rano) were determined from confluency at 72 h and for RT by clonogenic assay at 10 days post-treatment using a four-parameter logistic model. Symbols show mean survival (%) ± SEM for six replicates per experiment (*N* = 3). **(B)** Heat maps of the cell survival for TMZ/RT and DCA/Rano combinations. Black boxes represent the best synergy combination indices as calculated using the CompuSyn Software (Paramus, NJ, USA).

Gl261 was more TMZ-sensitive compared to CT2A (IC50; 2,526 vs. >10,000 μM) but less sensitive to DCA (25.9 vs. 16.0 mM), Rano (766.2 vs. 423.9 μM), and RT (4.0 vs. 2.2 Gy; Figure 2A). Comparative IC50 for human immortalized GBM cell lines U87-MG, U251-MG, and T98G is provided in Supplementary Figure 1. Synergistic drug concentrations for TMZ/RT and DCA/Rano were determined using the CompuSyn Software (Paramus, NJ, USA) (25, 26). Combination indices <1 indicate synergy, where 0 is additive and >1 antagonistic. The combination indices indicated that the synergistic doses for Gl261 were 39 μM TMZ/2 Gy RT and 37.5 mM DCA/156 μM Rano and for CT2A were 19.5 μM TMZ/1 Gy RT and 37.5 mM DCA/39.06 μM Rano (Figure 2B).

### DCA and Rano Energy Metabolism Assays Confirm Inhibition at Syngeneic Doses

**Figure 3:**
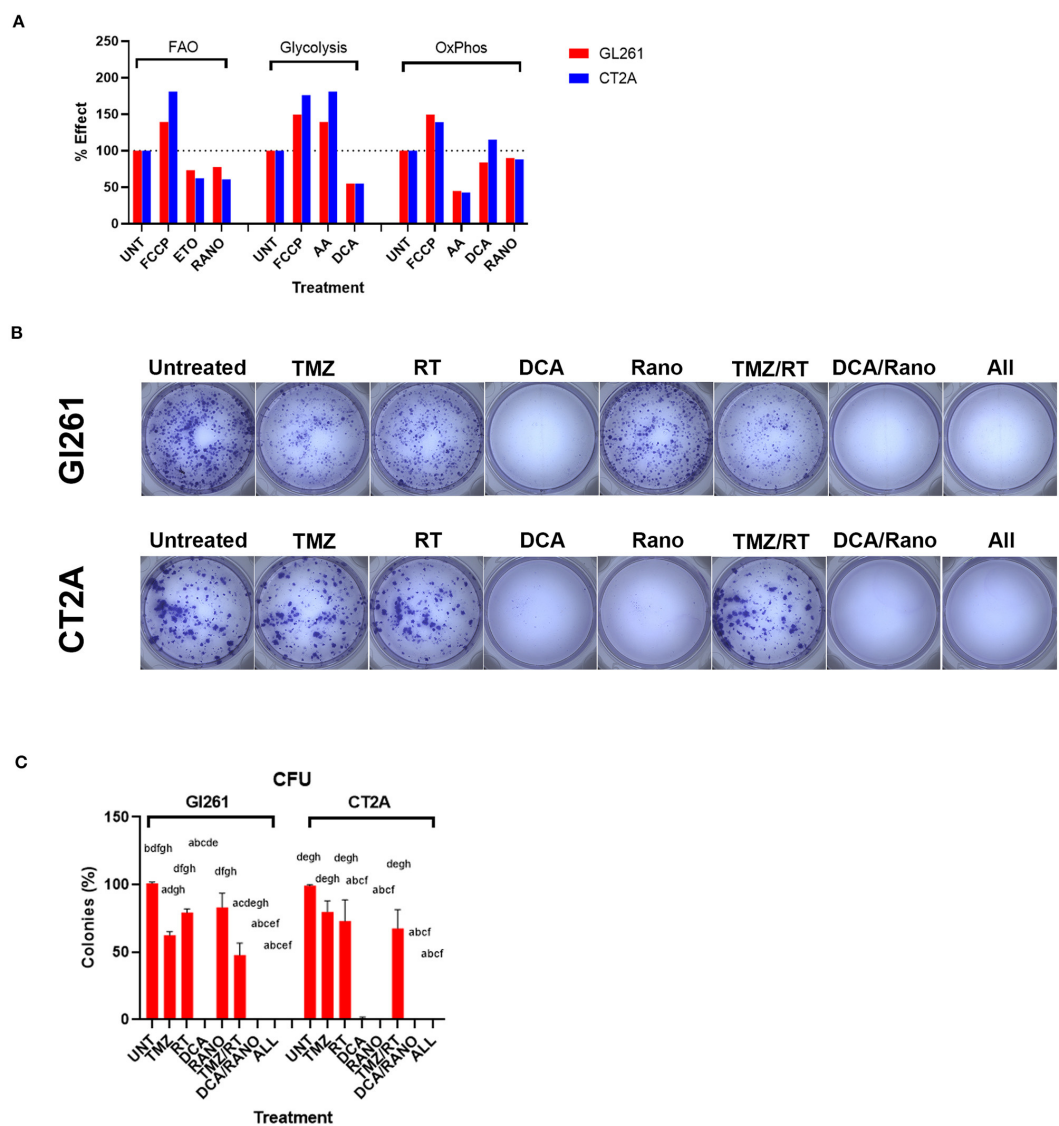
Temozolomide (TMZ), RT, DCA, and their combination reduce GBM cell proliferation. **(A)** Percentage effect of dual-read time-resolved fluorescent lifetime at 60-min post-treatment relative to untreated control (Unt) for fatty acid oxidation (FAO) and glycolytic and oxidative phosphorylation (OxPhos) metabolism. Carbonyl cyanide-4-(trifluoromethoxy)phenylhydrazone (FCCP; 2.5 μm), etomoxir (Eto; 40 μm), and antimycin A (AA; 1 μm) were used as positive and negative controls where appropriate. Data are expressed as mean of triplicates. **(B)** Micrographs of colonies (>50 cells) 10-day post-treatment. **(C)** Column graphs show mean ± SEM of triplicates from three independent experiments. Column graphs show the mean ± SEM from three independent experiments ^a^*p* < 0.05 vs. Unt; ^b^*p* < 0.05 vs. TMZ; ^c^*p* < 0.05 vs. RT; ^d^*p* < 0.05 vs. DCA; ^e^*p* < 0.05 vs. ranolazine **(**Rano); ^f^*p* < 0.05 vs. TMZ/RT; ^g^*p* < 0.05 vs. DCA/Rano; and ^h^*p* < 0.05 vs. All-combined treatments were determined by Kruskal–Wallis test alongwith the Dunn's multiple comparison test.

To confirm that DCA and Rano synergistic drug doses were sufficient to (partially) inhibit glycolytic and FAO metabolism, respectively, we performed dual-read time-resolved fluorescent assays. These assays measure the rate of fluorescent decay by providing a more stable and greater dynamic range than the traditional signal intensity measurements. Fluorescence lifetime was calculated as described in “Energy Metabolism Assays” section, and data were expressed relative to untreated controls (Unt). Each drug decreased its respective metabolic pathway by 23–45% at 60 min post-treatment (Figure 3A).

To assess the delayed impact on cell growth/survival, CFU assays were performed (Figure 3B). Using the synergistic doses identified in Figure 2B, DCA, Rano, and their combination significantly reduced colony formation compared to Unt (all *p* < 0.0001; Figure 3C), indicating no escape from the treatment control or emergence of resistance. In contrast, TMZ and RT alone and in combination with reduced CFU ~50% showed incomplete lethality. This suggests that DCA/Rano metabolic targeting is a viable strategy that did not induce a resistant population. We next sought to investigate potential chemo-radio-enhancement in oxidative stress, DNA damage, cell cycle, autophagy, and apoptotic mechanisms.

### DCA/Rano Increases ROS Levels in Murine GBM Cells

**Figure 4:**
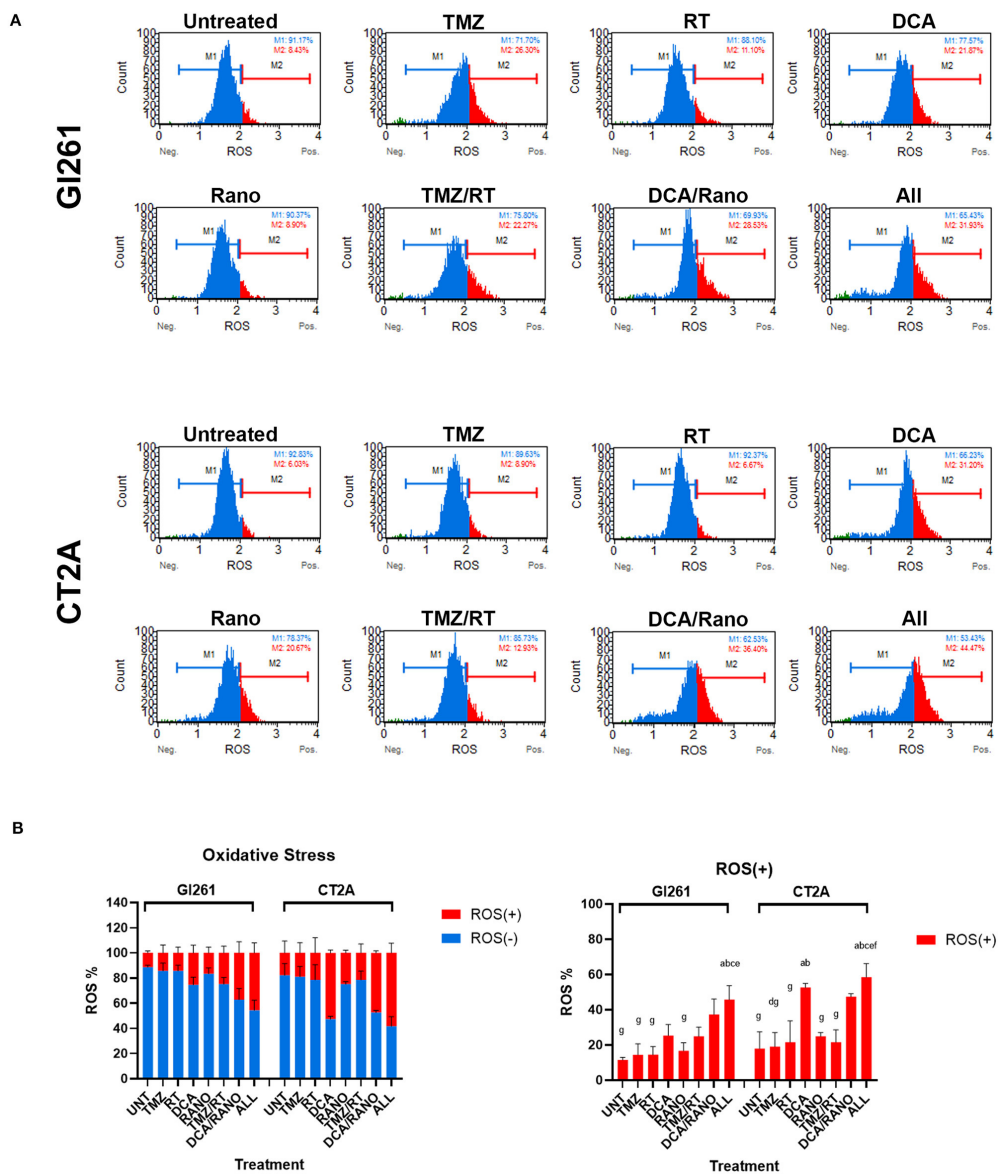
DCA/ranolazine (Rano) increases reactive oxygen species (ROS) levels in murine GBM cells. **(A)** Representative Luminex® histograms (Luminex Corp., Austin, TX, USA) show the ROS fluorescent intensity as ROS^−^ (M1; blue) and ROS^+^ (M2; red) in Gl261 and CT2A GBM lines. **(B)** Column graphs showing the mean ± SEM from three independent experiments as in **(A)**. ^a^*p* < 0.05 vs. Unt; ^b^*p* < 0.05 vs. temozolomide (TMZ); ^c^*p* < 0.05 vs. radiation therapy (RT); ^d^*p* < 0.05 vs. DCA; ^e^*p* < 0.05 vs. Rano; ^f^*p* < 0.05 vs. TMZ/RT; and ^g^*p* < 0.05 vs. All-combined treatments were determined by the two-way ANOVA test along with the Tukey's multiple comparison test.

The ability of RT to induce DNA damage is dependent on the generation of ROS (*via* radiolysis of water in cells). Inhibition of glycolysis and FAO by DCA and Rano, respectively, should increase the dependency of GBM cells on oxygen, thus increasing the ROS levels. In both cell lines, DCA and DCA/Rano induced an increase in ROS levels at 72-h post-treatment (Figure 4A). However, this was not significantly greater than TMZ/RT (Figure 4B). While all-combined treatments induced ROS generation, DCA was the primary single-treatment contributor, indicating that it may lead to increased DNA damage.

### DCA and TMZ Increase DNA Damage in Murine GBM Cells

**Figure 5:**
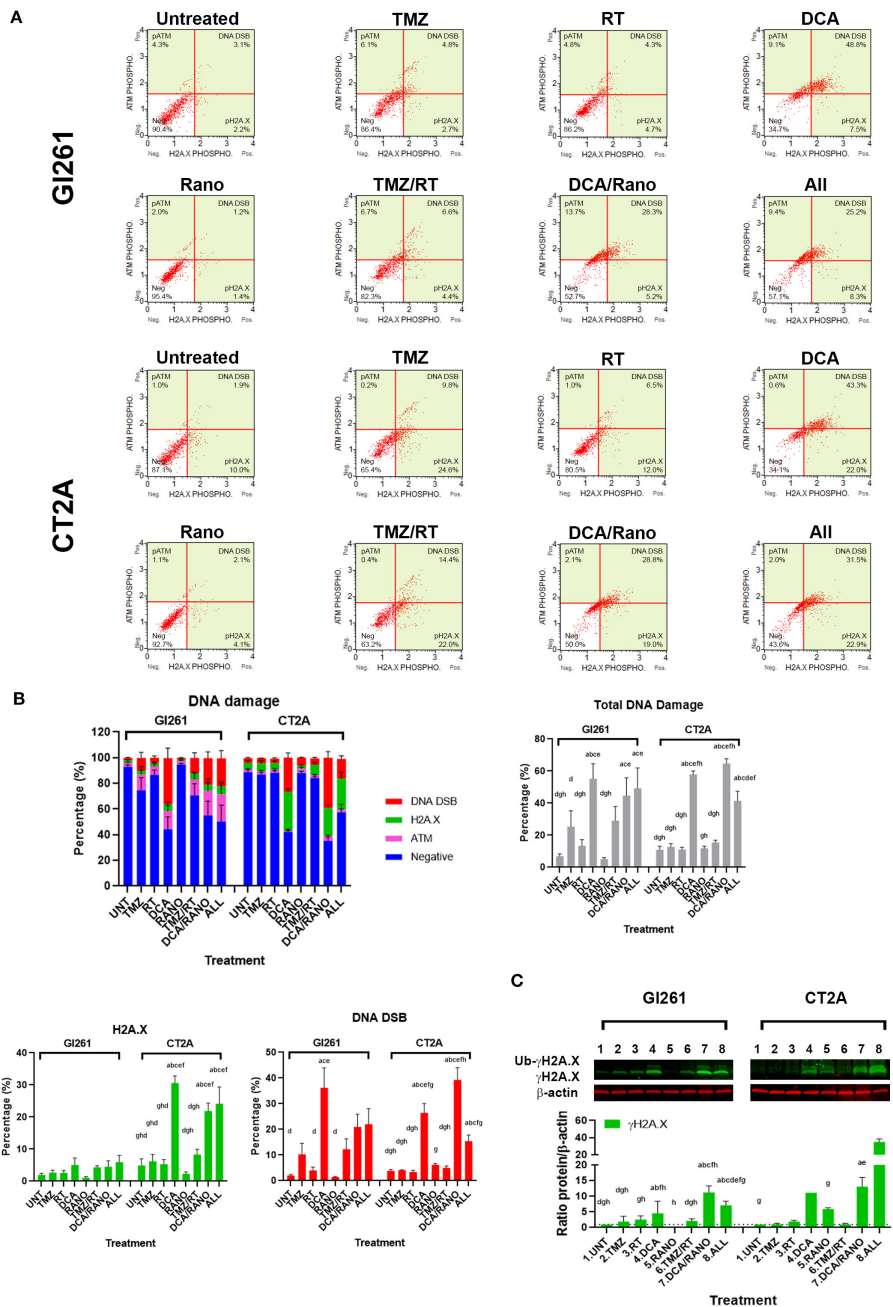
TMZ and DCA increase DNA damage in GBM cell lines. **(A)** Representative Luminex® dot plots (Luminex Corp., Austin, TX, USA) depict increased phosphoryated ataxia telangiectasia mutated (ATM) (upper left), phosphoryated H2A.X (lower right), or both (DSB; upper right). **(B)** Column graphs show mean ± SEM from three independent experiments as in **(A)**. ^a^*p* < 0.05 vs. Unt; ^b^*p* < 0.05 vs. TMZ; ^c^*p* < 0.05 vs. RT; ^d^*p* < 0.05 vs. DCA; ^e^*p* < 0.05 vs. Rano; ^f^*p* < 0.05 vs. TMZ/RT; ^g^*p* < 0.05 vs. DCA/Rano; and ^h^*p* < 0.05 vs. All-combined treatments were determined by the two-way ANOVA test along with the Tukey's multiple comparison test. **(C)** Western blot immunoblot (top) and densitometric analysis (bottom) of γH2A.X (S139) and β-actin in Gl261 and CT2A GBM cell lines (*N* = 5). The protein expression was normalized to reference the protein β-actin expression.

To determine whether the increased ROS levels led to increased DNA damage, phosphorylation of ATM (pATM) and H2A.X (pH2A.X) were assessed as early indicators of DNA double-strand breaks (DSB; Figure 5A). In both cell lines, DCA alone and DCA/Rano significantly increased total DNA damage to ~6-fold compared to Unt (*p* = 0.0059) and above that for TMZ/RT (CT2A; *p* < 0.0001). In Gl261, this was reflected in increased H2A.X phosphorylation and ATM activation (DNA DSB) at 72 h, whereas CT2A cells showed predominantly increased H2A.X phosphorylation (Figure 5B). Western blot of γH2A.X protein expression confirmed upregulation at 72-h post-treatment (Figure 5C).

### DCA and Rano Do Not Significantly Alter Cell Cycle Progression

**Figure 6:**
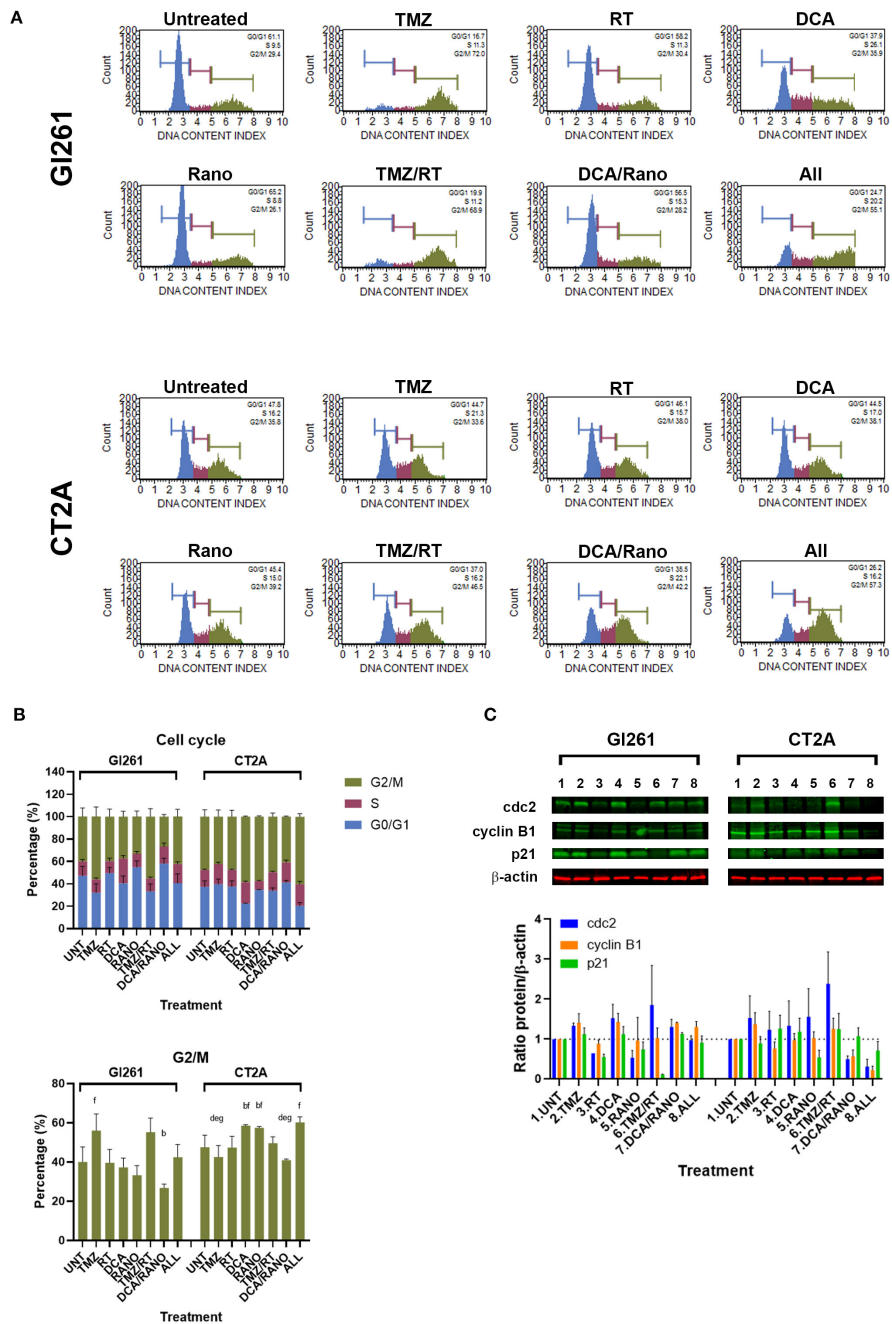
TMZ alone or combined with RT arrests Gl261 cells in the G2/M phase. **(A)** Representative Luminex® cell cycle histograms (Luminex Corp., Austin, TX, USA) show fluorescent intensity as the G0/G1 phase in blue, the S phase in purple, and the G2/M phase in green. **(B)** Column graphs show mean ± SEM from three independent experiments as in **(A)**. ^a^*p* < 0.05 vs. Unt; ^b^*p* < 0.05 vs. TMZ; ^c^*p* < 0.05 vs. RT; ^d^*p* < 0.05 vs. DCA; ^e^*p* < 0.05 vs. Rano; ^f^*p* < 0.05 vs. DCA/Rano; and ^g^*p* < 0.05 vs. All-combined treatments were determined by the two-way ANOVA test along with the Tukey's multiple comparison test. **(C)** Western blot immunoblot (top) and densitometric analysis (bottom) of the expression of cyclin-dependent kinase 2 (cdc2), cyclin B1, p21, and β-actin in GBM cell lines following treatment (*N* = 5). The protein expression was normalized to reference protein β-actin expression.

The cell cycle contains several checkpoints to prevent the proliferation of cells with DNA damage. To determine the effect of DCA and Rano on cell cycle, the percentage of cells in non/early-dividing (G0/G1), synthesis (S), and late-dividing/mitosis (G2/M) were assessed (Figure 6A). Treatment of Gl261 with TMZ or TMZ/RT led to an increase of 40% of cells in the G2/M phase, whereas the same treatment in CT2A had no effect (Figures 6A,B). This is consistent with an observed reduction in the size of individual TMZ and RT-treated colonies for Gl261 but not CT2A colonies in Figure 3B. TMZ/RT also showed a trend toward upregulated Cdc2, which is necessary for S/G2 and G2/M transition, but not cyclin B1 or p21 (Figure 6C). However, in both cell lines, the DCA/Rano reduced G2/M arrest, with a trend toward greater S-phase arrest, but did not reach significance.

### DCA and DCA/Rano Induce Autophagy in GBM Cells Lines

**Figure 7:**
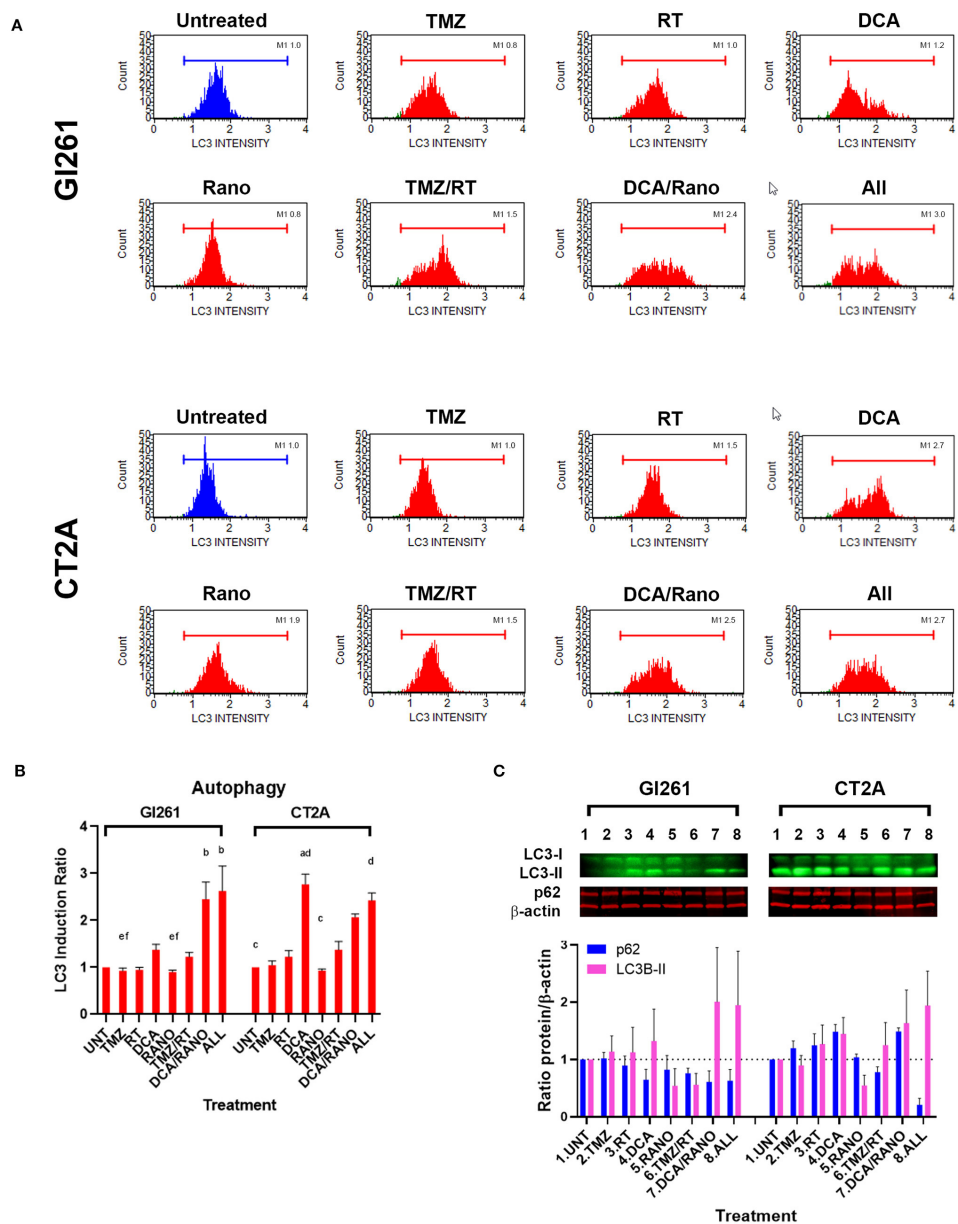
TMZ/RT and DCA/Rano induce autophagy in GBM cell lines. **(A)** Representative Luminex® histograms (Luminex Corp., Austin, TX, USA) of LC3 fluorescent intensity in Unt (blue) and treatment (red). **(B)** Column graphs show mean ± SEM from four independent experiments as in **(A)**. ^a^*p* < 0.05 vs. Unt; ^b^*p* < 0.05 vs. TMZ; ^c^*p* < 0.05 vs. DCA; ^d^*p* < 0.05 vs. Rano; ^e^*p* < 0.05 vs. TMZ/RT; and ^f^*p* < 0.05 vs. All-combined treatments were determined by the Kruskal–Wallis test along with the Dunn's multiple comparison test. **(C)** Western blot immunoblot (top) and densitometric analysis (bottom) of the expression of p62, LC3-II, and β-actin in GBM cell lines following treatment as indicated (*N* = 5). The protein expression was normalized to reference protein β-actin expression.

**Figure 8:**
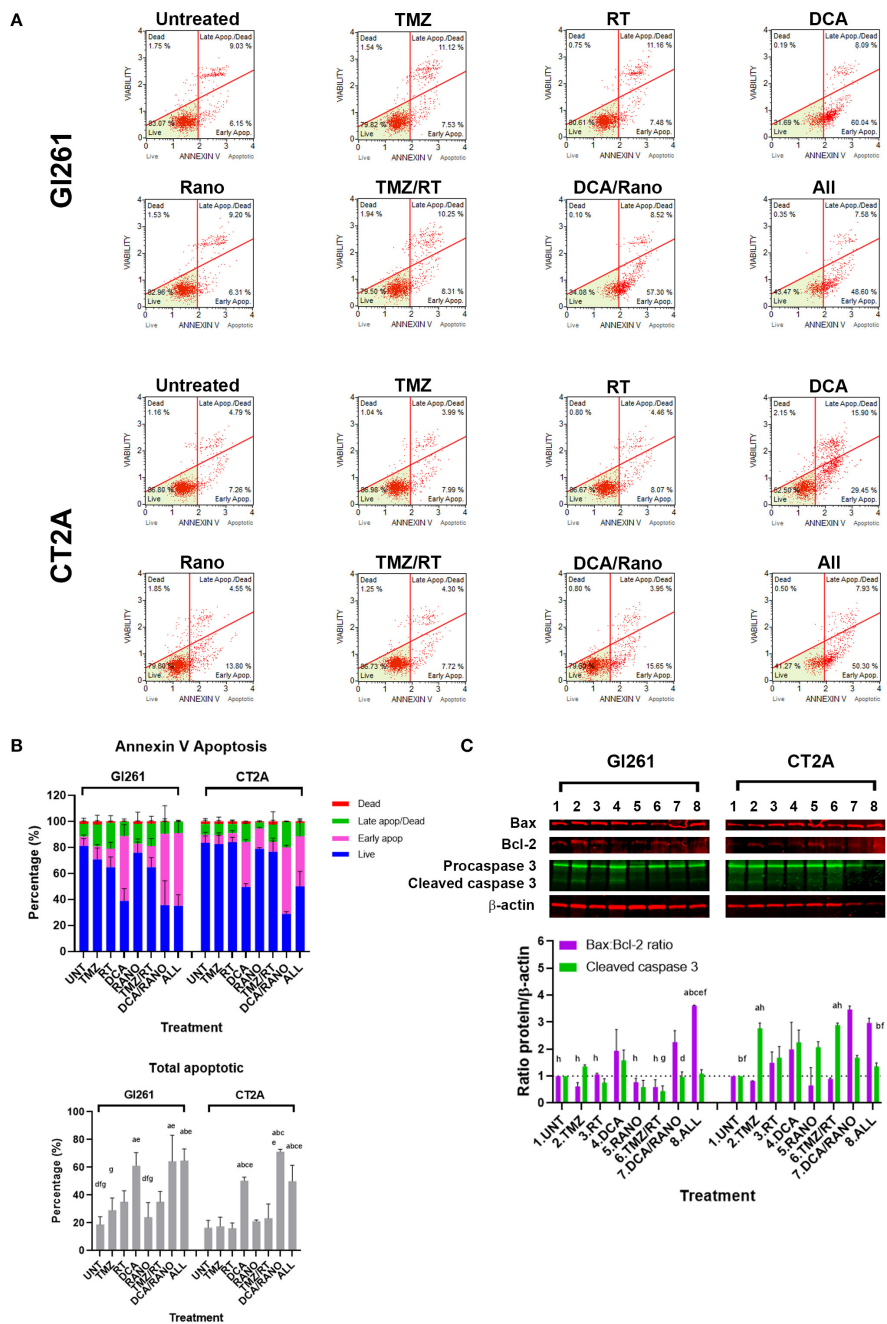
DCA alone or in combination increases early apoptosis in GBM cell lines. **(A)** Representative Luminex® dot plots (Luminex Corp., Austin, TX, USA) show live (lower left) annexin V(AV–)/7AAD(–) cells, early apoptotic (lower right) AV(+)/7AAD(–) cells, late apoptotic (upper right) AV(+)/7AAD(+) cells, and dead cells (upper left) AV(–)/7AAD(+) cells. **(B)** Column graphs show mean ± SEM from three independent experiments as in **(A)**. ^a^*p* < 0.05 vs. Unt; ^b^*p* < 0.05 vs. TMZ; ^c^*p* < 0.05 vs. RT; ^d^*p* < 0.05 vs. DCA; ^e^*p* < 0.05 vs. Rano; ^f^*p* < 0.05 vs. DCA/Rano; and ^g^*p* < 0.05 vs. All-combined treatments were determined by the two-way ANOVA test along with the Tukey's multiple comparison test. **(C)** Western blot immunoblot (top) and densitometric analysis (bottom) of the expression of Bax, Bcl-2, caspase 3, and β-actin in GBM cell lines following treatment (*N* = 5). The protein expression was normalized to reference protein β-actin expression.

DNA damage-induced autophagy can delay apoptotic cell death by mediating the degradation of specific cell cycle proteins, regulation of cell division, and promotion of DNA damage repair. To determine whether DCA and Rano induce autophagy, the expression of markers of the autophagosomal membrane, microtubule-associated protein light chain 3-II (LC3), was assessed by Luminex® assay (Luminex Corp., Austin, TX, USA), and Western blotting. In Gl261 cells, DCA/Rano, and all-combined treatments induced the expression of LC3 (Figure 7A) and 2.5-fold greater that LC3 induction of autophagy compared to Unt (*p* < 0.0001) (Figure 7B). This was accompanied by a decrease in the autophagy substrate p62 at 72-h post-treatment (Figure 7C), though it did not reach significance. Similarly, in CT2A cells, DCA, DCA/Rano, and all-combined treatments showed a trend toward a 2-fold increase in LC3-autophagic induction (*p* = 0.0211; Figure 7B). In CT2A cells, DCA, and DCA/Rano showed increased p62 levels (Figure 7C). Although p62 is recognized for its role in autophagic flux, it also has roles in the anti-oxidative stress response, nutrient sensing, and apoptosis (28), consistent with the increased ROS (Figure 4B) and the Bax:Bcl-2 ratio (Figure 8C) noted in CT2A cells. These results suggest that DCA induces autophagy in GBM cells but may be more effective when combined with Rano in Gl261 cells, as observed in the Gl261 DCA/Rano and all-combined groups.

### DCA Alone or Combined Increases Apoptosis in GBM Cell Lines

If GBM cells fail to overcome the increased ROS, DNA damage, cell cycle arrest, and autophagy, they will finally undergo apoptotic cell death. To assess the levels of apoptosis in Unt Gl261 and CT2A, the membrane-impermeable DNA dye 7-aminoactinomycin (7-AAD) and Annexin-V were assessed by Luminex® assay (Luminex Corp., Austin, TX, USA; Figure 8A). In DCA, DCA/Rano, and all-combined treatments, the total number of apoptotic cells increased 2- to 3-fold compared to Unt (*p* = 0.0273; Figure 8B). In both cell lines, the total apoptosis was mostly manifested in increased early apoptotic events [annexin-V^+^/7-AAD^**−**^ (viability); Figure 8B]. Consistent with the CFU data (Figure 3B), TMZ/RT did not significantly induce apoptosis (Figure 8B). To assess the nature of the apoptotic signal, we determined the expression of early proapoptotic protein Bax and anti-apoptotic protein Bcl-2 and late apoptotic cleaved caspase 3 (Figure 3C) by immunoblotting. In both cell lines, the Bax:Bcl-2 ratio, and caspase 3 in CT2A were increased by DCA, DCA/Rano, and all-combined treatments (Figure 8C), suggesting that DCA induces the mitochondrial (intrinsic) apoptotic pathway in GBM cell lines. In CT2A cells, TMZ, and TMZ/RT increased caspase 3 in the absence of increased annexin V and Bax:Bcl-2 (Figure 8C). For explanation, see “Discussion” section.

### DCA/Rano Increases *in vivo* Murine GBM Survival

**Figure 9:**
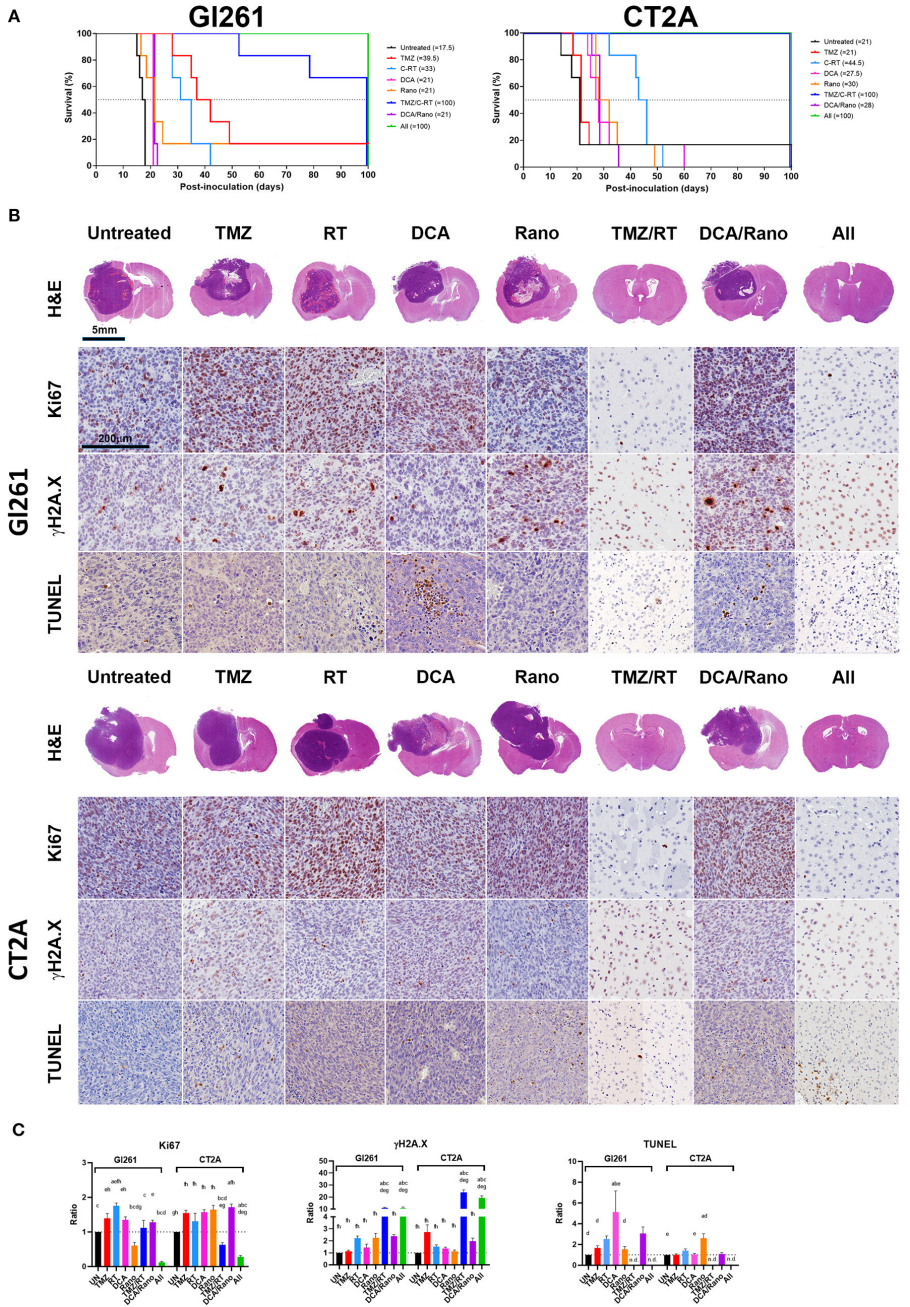
DCA and Rano increased median survival but were not more efficacious than chemoradiation. Mice were injected intracranially with 1 × 10^5^/2 μl murine GBM cells on day 0 and TMZ (50 mg/kg/day i.p.), DCA (200 mg/kg/day i.p.), Rano; 50 mg/kg/day i.p.), and/or RT (20 Gy/10) treatment administered daily from day 7 to day 21. **(A)** The Kaplan–Meier plots show median survival per treatment and are indicated in the graph legend. Six mice per treatment were monitored for 100-day postinoculation or until humane endpoint. **(B)** Endpoint Gl261 (top) and CT2A (bottom) tumors were immunohistochemically stained for cell proliferation marker, Ki67, DNA damage marker, γH2A.X (S139), and apoptotic marker, terminal deoxynucleotidyl transferase dUTP nick end labeling (TUNEL) stain. **(C)** Column graphs of the quantitation of positively stained cells in treated mice compared to controls. Data are expressed as mean ± SEM for five high-power fields (*N* = 6 tumors per treatment group). ^a^*p* < 0.05 vs. Unt; ^b^*p* < 0.05 vs. TMZ; ^c^*p* < 0.05 vs. RT; ^d^*p* < 0.05 vs. DCA; ^e^*p* < 0.05 vs. Rano; ^f^*p* < 0.05 vs. TMZ/RT; ^g^*p* < 0.05 vs. DCA/Rano; and ^h^*p* < 0.05 vs. All-combined treatments were determined by the two-way ANOVA test along with the Tukey's multiple comparison test. n.d., not determined.

Our *in vitro* data shows that DCA induces ROS generation, DNA damage, autophagy, and apoptosis, whereas, the effects of Rano were minimal. Some of the effects of Rano treatment have been shown to affect the immune and tumor microenvironment and the function of normal astrocytes and neurons (12, 13). To determine the clinical effect of Rano and combination treatments, we use two orthotopic syngeneic murine models (Supplementary Figure 2). Mice were injected intracranially with 1 × 10^5^/2 μl Gl261 or CT2A cells on day 0 and TMZ (50 mg/kg/day i.p.), DCA (200 mg/kg/day i.p.), Rano (50 mg/kg/day i.p.), and/or RT (20 Gy/10) treatment were administered daily from day 7 to 18. For both Gl261 and CT2A tumor models, DCA, Rano, and DCA/Rano significantly increased survival; up to 20% in Gl261 tumor-bearing mice (median survival; 21 vs. 17.5 days; *p* < 0.0001) and 40% in CT2A tumor-bearing mice compared to Unt tumor-bearing controls (29.5 vs. 21 days; *p* < 0.0001; Figure 9A). No significant differences in toxicity (as assessed by weight of animals) between treatments were observed (data not shown). Consistent with our *in vitro* IC50 data (Figure 2A), the median survival for CT2A tumor-bearing mice demonstrated greater TMZ resistance and RT sensitivity than Gl261 tumor-bearing mice (Figure 9A). The TMZ/RT combination in both models was superior to DCA/Rano with 50% median survival, thus not reaching the 100-day period (indicating long-term survival).

The histopathological analysis of the tumor morphology revealed that DCA and DCA/Rano induced focal regions of necrosis and hemorrhage with increased Ki67 proliferation indices and DNA fragmentation (cell death; TUNEL staining) in Gl261 tumors (Figure 9B); Rano significantly decreased Ki67 in Gl261 tumors but increased TUNEL in CT2A tumors. Tumors treated with TMZ and/or RT and all-combined treatment showed increased DNA damage (γH2A.X; *p* < 0.001; Figure 9C). We have previously observed this phenomenon of prolonged γH2A.X at 28 days after RT treatment in noncancerous brain, lung, and colon tissues (24). As the patterning is not observed in unirradiated (untreated) brains, we believe that it indicates radiotoxicity to the “healthy” cells, including radiosensitive oligodendrocytes, which have shown limited regenerative capacity out to 18-month postirradiations (29). In brain tissue where tumors resolved, TUNEL staining was limited to immune cells present along the wound tract (Figure 9B) and therein not quantitated.

## Discussion

Contrary to a recent report by Duraj and colleagues (27), we show that glucose and FAO metabolic pathways are similar among GBM subtypes, and enzymes of the glycolytic and FAO pathways are upregulated in GBM tumors compared to normal brain tissues. While many therapies aim to target the genetic differences in GBM tumors (e.g., EGFR, IDH, FGFR, and TACC), the inhibition of GBM cellular energetics is a potentially wide ranging approach with impact irrespective of GBM subtype.

Chemoradiation forms part of the “standard of care” for patients with GBMs; therefore, we compared the effects of DCA, Rano, and DCA/Rano in relation to chemoradiation and their known mechanism of action; ROS > DNA damage > cell cycle > apoptosis/autophagy, as well as *in vivo* efficacy. DCA and Rano induced oxidative stress, DNA damage, autophagy, and apoptosis (summarized in Supplementary Figure 3). At 72 h, the CT2A cells had lower ROS levels and less ATM activation compared to Gl261 cells. The subtle disparity in the response of Gl261 and CT2A cells may be partially attributable to differences in TP53 with Gl261 (P53^MUT^) and CT2A (P53^WT^) (30). TP53 is one of the most commonly dysregulated genes in GBM, with up to 54% of patients with TP53^MUT^ depending on the GBM subtype (1). However, it is reported that P53^WT^ inhibits lipid synthesis and glycolysis in normal and tumor cells, whereas T53^MUT^ promotes lipid synthesis and glycolysis (31). In tumor cells, it is not that simple. Not all P53 mutants increase glycolysis (32) and wild-type P53 can promote the metabolic switch from oxidative phosphorylation to glycolysis by inducing p53 upregulated modulator of apoptosis (PUMA)-mediated disruption of mitochondrial pyruvate uptake in cancer cells (33). Therein where we would have expected Gl261 (P53^MUT^) to be more sensitive to DCA, we instead observed higher IC50s for both DCA and Rano in the Gl261 line due to its precedence. In normal cells, wild-type P53 positively regulates ferroptosis, yet in tumor cells, TP53^MUT^ sensitizes tumor cells to ferroptosis, a process noted to have a role in TMZ resistance and is associated with GBM autophagy and apoptotic mechanisms (31). Further, examination of the P53 mutant and wild-type regulation of glycolytic and FAO pathways in GBM is needed.

One point of difference in our study was that in both cell lines, DCA increased annexin V, Bax:Bcl-2 ratio, and CT2A caspase 3, indicating that DCA induces the mitochondrial (intrinsic) apoptotic pathway in GBM cell lines. Yet, in CT2A, but not Gl261 cells, TMZ increased caspase 3 without increased annexin V or Bax:Bcl-2. A study by Roos et al. (34) proposed that, in glioma cells, O^6^-methylating agents, such as TMZ, induce the accumulation of DNA DSBs. In P53^WT^ cells (e.g., CT2A), this induction activates the extrinsic apoptotic pathway *via* FasR and caspase 8, whereas in P53^MUT^ cells (e.g., Gl261) the same response triggers the intrinsic apoptotic pathway *via* Bax:Bcl-2 and caspase 9. Both apoptotic pathways led to increased caspase 3 cleavage. Therein in our study, DCA and TMZ may trigger the intrinsic and extrinsic apoptotic pathways in CT2A, respectively, which was not observed in Gl261 cells due to differences in P53 status; however, we cannot account for the absence of membrane translocation for TMZ-treated CT2A cells in this study.

In line with other murine models examining glycolytic inhibitors, 2-DG, and/or FAO inhibitors, etomoxir or Rano, in GBM, lung and colon cancers, median survival, or tumor growth were minimally affected (11, 13). However, the combination strategy showed an enhanced antitumor effect (13). The observed 20–40% increase in median survival in syngeneic murine models using DCA/Rano (Gl261: 17.5 vs. 21 days, *p* < 0.0001 and CT2A: 21 vs. 28 days, *p* < 0.0001) was comparable to an immunocompromised model of GBM (MES93) using 2-DG/etomoxir, which increased median survival from 17 to 24 days (*p* < 0.001) (11). It is noted that the effects of Rano on the cell may not be limited to FAO inhibition. Although, noted as a partial FAO inhibitor, Rano was found to lack the FAO-interfering activity in complete media with or without serum (35), despite showing FAO-inhibiting ability in our FAO assay utilizing glucose-deprivation and a single FAO source (18C unsaturated fatty acid oleate).

In GBM, DCA has shown promising results across a range of preclinical studies, yet these data have not translated into pronounced improvements in the few clinic trials undertaken thus far. Although, we demonstrated *in vitro* that DCA and Rano induced ROS, DNA damage, autophagy, and apoptosis, our *in vivo* survival data did not reflect this. *In vivo* dose, regimen, and sequencing of drugs may have limited therapeutic efficacy. In the present study, we administered DCA and Rano concurrently with chemoradiation. As most DNA damage from RT is due to indirect ionization of oxygen and increased ROS (peaking at ~30–120 min post-RT) and FDA clinical pharmacokinetic data available for TMZ, DCA, and Rano indicating that they reach peak plasma concentrations (*C*_max_) at 1–2 h, we elected to deliver RT at ~1-h post-drug administration. This contrasts phase I clinical trials in recurrent GBM, wherein, patients received DCA monotherapy two times daily for 30 days, without chemoradiation (36), and Rano monotherapy is FDA approved for administration two times daily for the treatment of angina. Further, elucidation of the best dose regimen and sequencing of the novel or repurposed therapeutics to target cancer cell metabolism will improve efficacy.

In GBM, the peripheral neuropathy of the patient from DCA has been noted, albeit transient and dose-dependent (15). The computational modeling reveals that the docking binding energy values of DCA are PDK2 > PDK1 > PDK4 > PDK3 (37), suggesting that DCA has greatest binding affinity for PDK2, for which we showed no significant difference in PDK2 gene expression between GBM tumors and normal brain tissue in the TCGA dataset. Of equal concern is the fact that *in vitro* concentrations of DCA are in the millimolar range, raising the notion that DCA derivatives, novel or repurposed drugs with selective PDK1/3 binding affinity, and micromolar or nanomolar IC50s may be better therapeutic avenues to explore.

In conclusion, the present study shows that dual glycolytic and FAO targeting with and without concomitant chemoradiation warrants further investigation in immunocompetent syngeneic models for GBM.

## Data Availability Statement

The datasets presented in this study can be found in online repositories. The names of the repository/repositories and accession number(s) can be found below: https://www.ncbi.nlm.nih.gov/geo/, GSE72217; https://www.ncbi.nlm.nih.gov/gap/, phs000178.

## Ethics Statement

The animal study was reviewed and approved by Northern Sydney Local Heath District Animal Ethics Committee, Royal North Shore Hospital, St Leonards, Australia.

## Author Contributions

KM, EW, MB, SS, AM, CD, and VH contributed to the conception and design of the study. KM performed the *in vitro* and *in vivo* experimentation and statistical analyses and wrote the first draft of the manuscript. All authors contributed to the manuscript revision and read and approved the submitted version.

## Conflict of Interest

The authors declare that the research was conducted in the absence of any commercial or financial relationships that could be construed as a potential conflict of interest.

## Supplementary Material

Supplementary Figure 1 Sensitivity and survival of murine and human glioma cell lines to drug and RT treatment combinations. Drug IC50s for TMZ, DCA, and Rano were determined from confluency (%) at 72 h, and RT by clonogenic assay at 10-day post-treatment using the four-parameter logistic model. Symbols show mean survival (%) ± SEM for six replicates per experiment (*N* = 3). U-87 MG did not form colonies in the RT assessment.

Supplementary Figure 2 Experimental timeline for Gl261 and CT2A tumor-bearing mice. Drugs and radiation therapy (RT) were assessed individually and in combination; RT 2 Gy/10, 50 mg/kg i.p. TMZ, 200 mg/kg i.p. DCA, and/or 50 mg/kg i.p. Rano five times per week for 2 weeks commencing at day 7 post-intracranial injection of 1 × 10^5^/2 μl Gl261 or CT2A cells. Mice were euthanized at humane endpoints or at day 100 postinoculation (considered long-term survival).

Supplementary Figure 3 Schematic representation of DCA and Rano inhibition on murine GBM cell lines. Drugs and chemoradiation were assessed individually and in combination. PS, phosphatidylserine.
