# Supplementary figures and images for: Glycolysis and Fatty Acid Oxidation Inhibition Improves Survival in Glioblastoma
Source: Front Oncol. 2021 Mar 29;11:633210. doi: 10.3389/fonc.2021.633210 (PMC8039392; doi:10.3389/fonc.2021.633210)

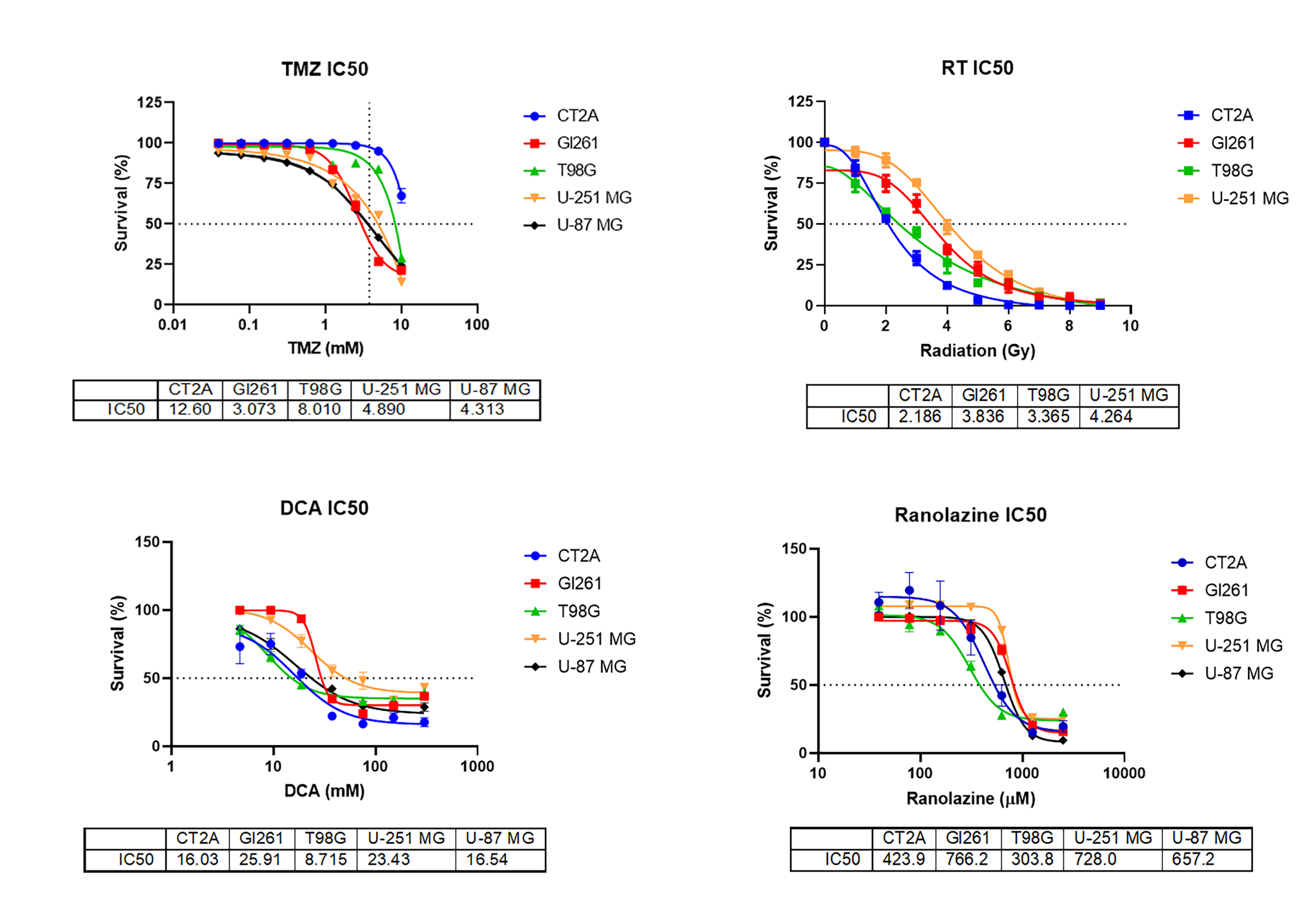

Supplement: Supplementary Figure 1 — Sensitivity and survival of murine and human glioma cell lines to drug and RT treatment combinations. Drug IC50s for TMZ, DCA, and Rano were determined from confluency (%) at 72 h, and RT by clonogenic assay at 10-day post-treatment using the four-parameter logistic model. Symbols show mean survival (%) ± SEM for six replicates per experiment (N = 3). U-87 MG did not form colonies in the RT assessment. [file Image_1.TIF]

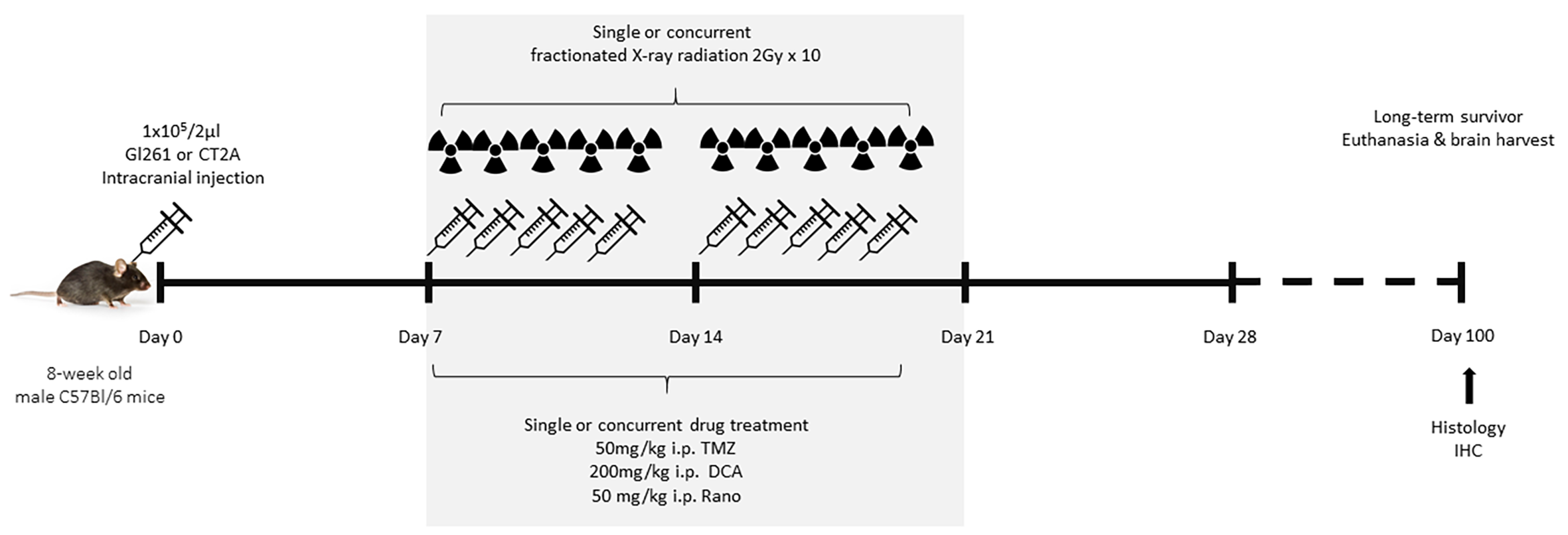

Supplement: Supplementary Figure 2 — Experimental timeline for Gl261 and CT2A tumor-bearing mice. Drugs and radiation therapy (RT) were assessed individually and in combination; RT 2 Gy/10, 50 mg/kg i.p. TMZ, 200 mg/kg i.p. DCA, and/or 50 mg/kg i.p. Rano five times per week for 2 weeks commencing at day 7 post-intracranial injection of 1 × 105/2 μl Gl261 or CT2A cells. Mice were euthanized at humane endpoints or at day 100 postinoculation (considered long-term survival). [file Image_2.TIF]

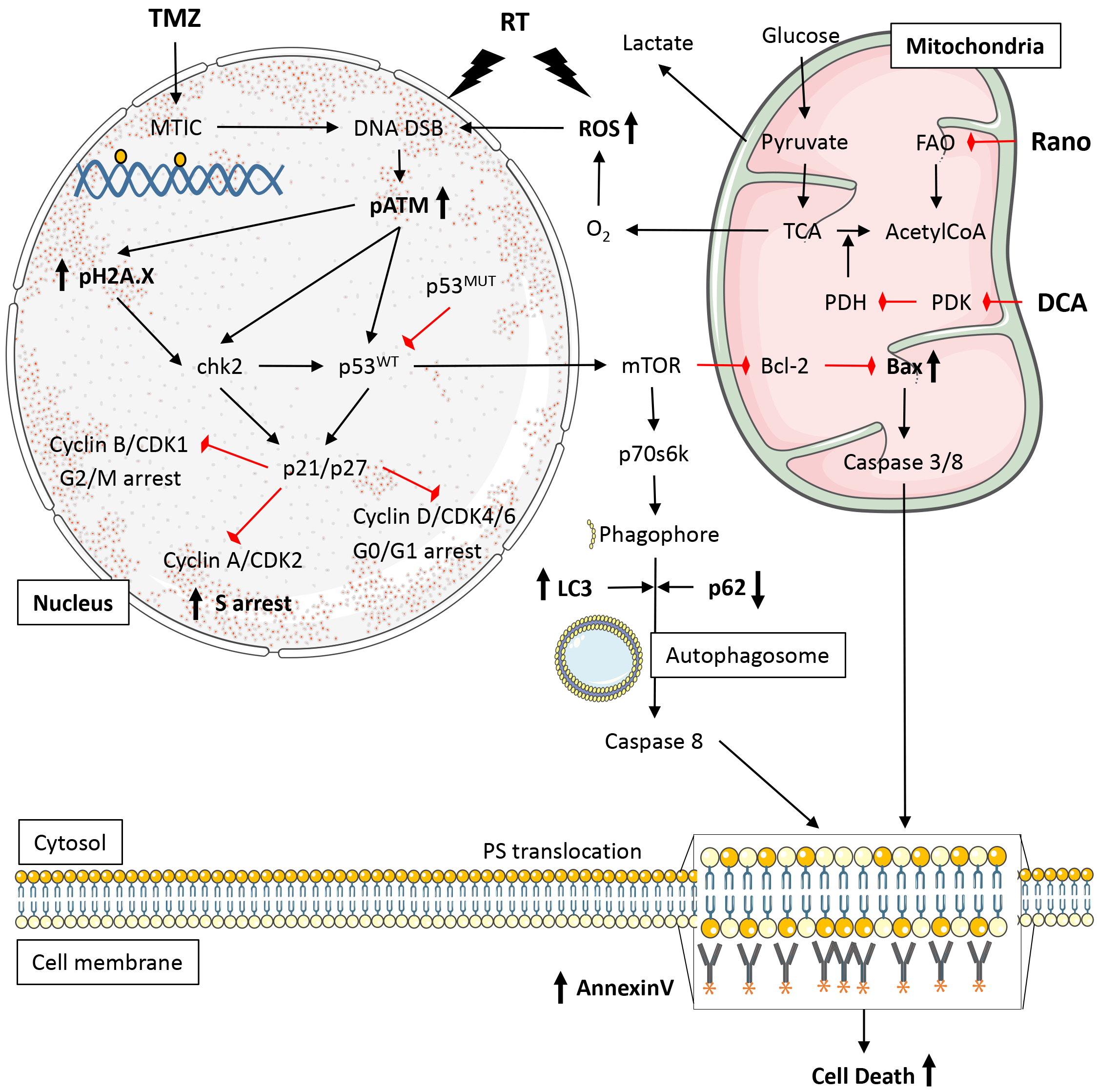

Supplement: Supplementary Figure 3 — Schematic representation of DCA and Rano inhibition on murine GBM cell lines. Drugs and chemoradiation were assessed individually and in combination. PS, phosphatidylserine. [file Image_3.TIF]
